# Pulsed Electric Field-Assisted “Green” Extraction of Betalains and Phenolic Compounds from *Opuntia stricta* var. *dillenii* Prickly Pears: Process Optimization and Biological Activity of Green Extracts

**DOI:** 10.3390/foods14172934

**Published:** 2025-08-22

**Authors:** Iván Gómez-López, Annachiara Pirozzi, Serena Carpentieri, María P. Portillo, Gianpiero Pataro, Giovanna Ferrari, M. Pilar Cano

**Affiliations:** 1 Laboratory of Phytochemistry and Plant Food Functionality, Biotechnology and Food Microbiology Department, Institute of Food Science Research (CIAL) (CSIC-UAM), Nicolás Cabrera 9, 28049 Madrid, Spain; ivan.gomez@ehu.eus; 2 Nutrition and Obesity Group, Department of Nutrition and Food Science, Faculty of Pharmacy, University of the Basque Country (UPV/EHU), Lucio Lascaray Research Center, 01006 Vitoria-Gasteiz, Spain; mariapuy.portillo@ehu.eus; 3 CIBERobn Physiopathology of Obesity and Nutrition, Institute of Health Carlos III (ISCIII), 01006 Vitoria-Gasteiz, Spain; 4 Department of Industrial Engineering, University of Salerno, Via Giovanni Paolo II, 132, 84084 Fisciano, Italy; apirozzi@unisa.it (A.P.); scarpentieri@unisa.it (S.C.); gpataro@unisa.it (G.P.); 5 BIOARABA Institute of Health, 01006 Vitoria-Gasteiz, Spain; 6 ProdAl Scarl–University of Salerno, Via Giovanni Paolo II, 132, 84084 Fisciano, Italy

**Keywords:** Pulsed Electric Fields (PEF), extraction, *Opuntia stricta* var. *dilenii*, betalains, phenolic compounds, antioxidant activity, anti-inflammatory activity

## Abstract

*Opuntia stricta* var. *dillenii* (OPD) fruits are rich in betalains and phenolic compounds, which are recognized for their potential health-promoting properties. This study focuses on the optimization of pulsed electric field (PEF)-assisted solid–liquid green extraction (SLE) from OPD whole fruit, using response surface methodology (RSM) experimental design to obtain green extracts rich in bioactive compounds. The optimal PEF pre-treatment conditions (electric field strength and energy input) were determined based on the cell disintegration index (Zp), followed by optimizing SLE conditions (temperature, time, and ethanol content). High-performance liquid chromatography (HPLC-DAD-ESI-Qtof) was used to characterize the individual bioactive compound profile of the obtained OPD green extracts. Results showed that optimal PEF pre-treatment conditions were at 10.5 kJ/kg and 5 kV/cm, followed by SLE at 35 °C for 165 min, using water as the solvent. Conventional optimal SLE conducted at 45 °C, 8% ethanol, and 128 min was applied as the control process. The combined PEF-assisted SLE process enhanced total betalain and phenolic compound yields by 61% and 135%, respectively. Antioxidant activities (DPPH by 145%, FRAP by 28%) and anti-inflammatory potential (hyaluronidase inhibition by 19%) were also significantly improved. This study underscores the potential use of a PEF pre-treatment to improve obtaining green extracts rich in bioactive compounds with high biological activities from OPD whole fruits, using water as a solvent.

## 1. Introduction

Nowadays, climate change has shifted temperatures and weather patterns, causing worldwide warming. That is why some fruits, such as prickly pears, are becoming popular due to their adaptability to arid and semiarid climates. These fruits belong to the most abundant genus within the *Cactaceae* family (*Opuntia* spp.) [1]. *Opuntia ficus-indica* is the most commercialized, consumed, and investigated species [2]; however, there are a few others that are growing wildly, such as *Opuntia stricta* var. *dillenii* (OPD). In addition to being a wild species, OPD represents an underutilized green biomass resource, as it has been less investigated and may be of great interest to the food and pharmaceutical industries due to its nutritional properties and supposed benefits in preventing chronic diseases [3,4,5,6]. Indeed, prickly pears had been used in folk medicine [7] due to their rich source of bioactive compounds such as betalains and phenolic compounds, possessing health-promoting properties [8,9,10].

OPD produces small fruit with a dark purple pulp and peel. The intense purple color indicates that OPD are a rich source of betalains, particularly betacyanins, a purple-colored nitrogen-based dye, especially betanin [11]. Additionally, OPD are also considered a significant source of phenolic compounds, namely phenolic acids, with piscidic acid being the most important of this kind of compound, and flavonoids, with isorhamenetin glucoxylrhamnosyl-pentoside (IG2) being the most abundant ones [2,12]. The use of underutilized wild varieties such as OPD aligns with current strategies aimed at the sustainable transformation of agro-industrial biomass into high-value food ingredients. This valorization potential is especially relevant given the rich composition of bioactive compounds (betalains and phenolic compounds) in OPD, which could be extracted and applied in functional food formulations or nutraceuticals.

These bioactive compounds could be extracted from the plant matrix by different processes. For non-volatile compounds such as betalains and phenolic compounds, the oldest extraction process is the solid–liquid extraction (SLE), which consists of putting the starting plant material into contact with a solvent (water, alcohol, oil, etc.) and mixing them by different methods [13]. Nevertheless, the plant cell structure imposes a considerable barrier to extracting the bioactive compounds, which are the betalains, which are localized in vesicles and vacuoles (in the cytoplasm), and phenolic compounds mainly linked to the cell wall [14]. For that reason, the SLE technique has several drawbacks, such as a long processing time, low selectivity, and a large amount of energy and solvent required [15]. In order to face these issues, new extraction methods have been proposed to assist SLE in producing cell disruption or permeabilization as pre-treatment technologies. These methods could enhance the extractability of target intracellular compounds from plant matrices while reducing solvent use, time, and energy consumption [16]. New methods for cell disruption, such as microwave extraction, pressure liquid extraction, and ultrasonic-assisted extraction, have demonstrated an increase in the extractability of bioactive compounds [17,18,19,20]. Nonetheless, electro-technologies such as pulsed electric fields (PEF) have been shown to be promising as a milder and more efficient alternative to conventional cell disintegration techniques [21]. This non-thermal process reduces the degradation of heat-sensitive compounds that might occur in traditional extraction techniques involving high temperatures. Additionally, PEF extraction typically requires shorter processing times and lower energy consumption, making it a more environmentally friendly and cost-effective alternative [22,23].

PEF pre-treatment consists of the exposure of biological cells to repetitive, very short voltage pulses (typically from a few μs up to 1 ms) with an electric field intensity in the range of 0.5–10 kV/cm and an energy input that can vary from 0.5 to 20 kJ/kg. It has been proven that exposure to an electric field induces the permeabilization of cell membranes by electroporation, facilitating the release of valuable compounds from the inner parts of plant cells [21].

Moreover, to fully exploit the potential advantages arising from the application of PEF-assisted SLE compared to the conventional SLE process, an optimization step should be required. However, to date, only a few works have tested the applicability of PEF technology as a pre-treatment to intensify the juice yield and/or the recovery yield of bioactive compounds from *Opuntia ficus-indica* fruits [24] and *Opuntia stricta* var. *dillenii* fruits (OPD) [25,26,27], without focusing on the optimization of the extraction process made of a PEF pre-treatment followed by the SLE step. The application of PEF-assisted extraction could reduce energy and solvent use compared to conventional methods, contributing to more sustainable and resource-efficient processes. This extraction process may enable the extraction of bioactive compounds from OPD (betalains and phenolics), which possess antioxidant and anti-inflammatory activities and could be used as natural food colorants.

Therefore, the main aim of this study was to investigate the potential of PEF pre-treatment to enhance the extractability of bioactive compounds, such as betalains and phenolic compounds (phenolic acids and flavonoids), from OPD whole fruit puree to obtain green extracts with high antioxidant and anti-inflammatory activities. To achieve this, the study optimized the SLE process and the PEF-assisted process. To evaluate the effect of PEF pre-treatment on phenolic and betalain compounds, the green extracts obtained under optimized conditions were analyzed by high-performance liquid chromatography (HPLC).

## 2. Materials and Methods

### 2.1. Plant Material and Chemicals

**Figure 1:**
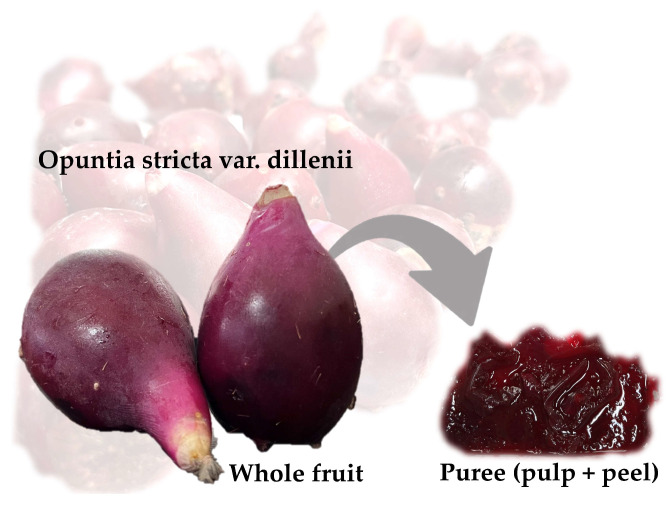
*Opuntia stricta* var. *dillenii* whole fruit and its puree.

*Opuntia stricta* var. *dillenii* prickly pears (OPD) were harvested on 20 May 2023, from Tinajo, Lanzarote, Canary Islands (Spain), at 205 m above sea level (29°03′ N, 13°40′ W). Fruits were selected based on size, peel coloration, and ripeness, discarding the damaged ones. After washing, fruits were characterized in terms of physicochemical properties (Table S1, Supplementary Materials), in accordance with Gómez-López et al. [12]. Before the extraction processes, the fresh OPD whole fruits were cut into slices, and these pieces were crushed to obtain a fruit puree (pulp and peels), removing the seeds by passing it through a 2 mm mesh sieve (Figure 1).

Solvents and chemical reagents were purchased from Sigma Aldrich (Steinheim, Germany). For characterization, standards were obtained in our laboratory. Betanin was purified from commercial beetroot using Sephadex L20 resin, and betaxanthins were semi-synthesized from the purified betanin. Phyllocactin was isolated from cactus berry fruits (*Myrtillocactus geometrizans*) using semi-preparative high-performance liquid chromatography (HPLC). Regarding phenolic compounds, piscidic acid was also purified from prickly pear peels using semi-preparative HPLC. Isorhamnetin glycoside standards were provided by Dr. Serna-Saldivar’s laboratory at the Biotechnology Center FEMSA (School of Engineering and Sciences, Tecnológico de Monterrey, Monterrey, Mexico).

For in vitro assays of biological activities, sodium chloride (NaCl), sodium acetate (C_2_H_3_NaO_2_), and sodium hydroxide (NaOH) were purchased from Panreac Quimica SLU (Barcelona, Spain). Sodium hyaluronidase was acquired from Acros Organics (Saddle Brook, NJ, USA). Sodium carbonate (Na_2_CO_3_), ascorbic acid (C_6_H_8_O_6_), iron(III) chloride hexahydrate (FeCl_3_·6H_2_O), 2,3,5-Triphenyl-2H-tetrazolium chloride (TPTZ), 1,1-diphenyl-2-picryl-hydrazil (DPPH), hexadecyltrimethylammonium bromide (CTAB) (C_19_H_42_BrN), and hyaluronidase enzyme were supplied by Sigma-Aldrich (St. Louis, MO, USA).

### 2.2. Pulsed Electric Fields (PEF) Equipment

PEF pre-treatments of OPD were carried out employing a batch PEF system previously described by Donsí et al. [28]. Briefly, the apparatus consisted of a treatment chamber made of two stainless steel parallel plate cylindrical electrodes (3 cm in diameter) separated by a polycarbonate tube (electrode gap up to 5 cm). A high-voltage cable and high-voltage pulsed power (25 kV − 500 A) generator (Modulator PG, ScandiNova, Uppsala, Sweden) connected to the treatment chamber allowed the delivery of monopolar square wave pulses with different pulse widths from 3 to 25 μs and frequencies from 1 to 450 Hz through the plant tissue. A high-voltage probe (P6015A, Tektronix, Wilsonville, OR, USA) and a Rogowski coil (Model 2 − 0.1, Stangenes Inc., Palo Alto, CA, USA), both connected to a 300 MHz oscilloscope (TDS 3034B, Tektronix, Wilsonville, OR, USA), were employed to record the actual voltage and current signals at the treatment chamber.

### 2.3. Determination of Cell Disintegration Index (Zp)

The cell disintegration index (Zp) was determined by impedance measurements according to the method described by Bobinaitė et al. [29] as an indicator of the degree of plant tissue (OPD fruit puree) disintegration induced by PEF pre-treatment. Measurements of the electrical complex impedance of untreated and PEF-treated samples were performed by placing 7.5 g of OPD puree into the treatment chamber. To ensure electrical continuity between the electrodes, 1 mL of MilliQ water was added. An impedance analyzer (Solartron 1260, Harrow, Middlesex, UK), operating in the frequency range of 10^2^–10^7^ Hz, was used.

### 2.4. Pulsed Electric Fields (PEF) Treatment

PEF pre-treatments were performed using a fixed pulse width of 20 μs and a frequency of 5 Hz, while varying the electric field strength (E = 1–5 kV/cm) and the specific energy input (WT = 1–20 kJ/kg), as derived from the experimental design reported in Table 1. During the treatments, no remarkable temperature increases were observed, as measured by a probe. The pH of the OPD puree was consistent across all studies, 3.82 ± 0.05 (Table S1). Electrical conductivity was not measured for each treatment, as all samples originated from the same homogenized batch with uniform physicochemical properties. Therefore, the initial conductivity was constant and not expected to influence the comparative analysis of Zp values.

The Zp value was calculated for each PEF treatment condition based on the measurement of the absolute value of the complex impedance |Z| of untreated (|Zuntr|) and treated tissue (|Ztr|) in the low (0.1 kHz) and high (1 MHz) frequency ranges [30].

The optimum electric field (Eopt, kV/cm) and energy input (WT-opt, kJ/kg) of the PEF pre-treatment were selected by identifying the condition that resulted in the highest Zp value, which indicates the most effective cell membrane permeabilization with the lowest treatment severity. This optimal condition was applied during the subsequent PEF-assisted extraction experiments.

### 2.5. Optimization of SLE and PEF-Assisted SLE Process

The optimization of PEF-assisted SLE was conducted in two steps: first, by the optimization of PEF pre-treatment based on the highest cell disintegration index (Zp) value; and second, solid–liquid extraction (SLE) conditions were optimized on pre-treated samples, focusing on extraction yield of bioactive compounds and the biological activities of the obtained extracts. Additionally, in order to measure the enhancement of the PEF pre-treatment, SLE with non-treated samples was optimized.

#### 2.5.1. SLE Process and PEF-Assisted SLE Process

The SLE process involved maceration of OPD whole fruit puree with the solvent in an orbital shaker, as derived from the experimental design, Table 2. Water–ethanol mixtures at different concentrations were added to the OPD samples at a constant prefixed solid-to-liquid ratio of 1:12 g/mL, following the method outlined by Carpentieri et al. [30]. The extraction process was performed in an orbital incubator S150 (PBI International, Milan, Italy), where the flasks were placed under continuous agitation at 160 rpm, with varying durations (30–300 min) and temperatures (20–50 °C), as detailed in Table 2. After that, the samples were centrifuged with the PK130R model (ALC International, Venaria Reale, TO, Italy) at 5289 x g for 10 min at 4 °C to separate the supernatants. Ethanol was eliminated from the obtained supernatants with an evaporator (Buchi, Flawil, Switzerland) at 25 °C, and later the remaining extracts were made up to 10 mL with MilliQ water. The obtained extracts were immediately frozen and stored at −24 °C until further analyses.

To conduct the PEF-assisted SLE experiments, 7.5 g of OPD fruit puree were loaded into the treatment chamber and treated with PEF under the previously defined optimal conditions (Eopt, WT-opt). After the electro-permeabilization treatment, the same SLE process was applied, according to the design shown in Table 2.

#### 2.5.2. Experimental Design

A two-factor face-centered central composite design (FC-CCD), consisting of 9 runs, including 2 central points and 2 factorial and 2 axial points (Table 1), was used to investigate the effects of the electric field strength (E) (X1, 1–5 kV/cm) and total specific energy input (WT) (X2, 1–20 kJ/kg) on the cell disintegration index, Zp (Yk), of OPD induced by PEF.

A second polynomial model reported in Equation (1) was used to predict the response variable, namely the cell disintegration index of PEF-treated samples (Zp), as a function of the independent variables.

(1) $$Y_{k}=\beta_{0}+\sum_{i=1}^{2}\beta_{i}X_{i}+\;\sum_{j=1}^{2}\beta_{ii}X_{i}^{2}\;+\;\sum_{i=1}^{2}\sum_{j=i+1}^{3}\beta_{ij}X_{i}X_{j}$$

where *Yₖ* represents the response variable; *Xᵢ* and *Xⱼ* denote the independent variables; and *β*_0_, *βᵢ*, *βᵢᵢ*, and *βᵢⱼ* correspond to the intercept and the regression coefficients for the linear, quadratic, and interaction terms of the model, respectively.

The same experimental model (FC-CCD), consisting of 12 runs (Table 2), was used to optimize the SLE process for both untreated (control) and PEF pre-treated OPD. The experimental runs were randomized, and two independent replications were performed for each combination to reduce the impact of unexpected variability. A second polynomial model (as Equation (1)) was used to predict the response variable, namely TPC, FC, total betacyanins, total betaxhantin, antioxidant activity, and anti-inflammatory activity of untreated and PEF pre-treated samples as a function of the independent variables, namely temperature (X3, 20–50 °C), time (X4, 30–300 min), and EtOH content in water (X5, 0–100% *v*/*v*).

### 2.6. Characterization of SLE and PEF-Assisted SLE OPD Extracts

#### 2.6.1. Total Phenolic Compounds (TPC) Analysis

The Folin–Ciocalteau method was used to determine the TPC of obtained OPD extracts following the methodology reported by Bobinaitė et al. [29]. The absorbance of the reacting mixture was measured at 765 nm using a UV/Vis spectrophotometer (V-650, Jasco Inc., Easton, MD, USA). A gallic acid standard calibration curve was used to quantify, and the TPC concentration was expressed as milligrams of gallic acid equivalents (mg GAE) per gram of dry weight (g DW) of OPD.

#### 2.6.2. Total Flavonoid (FC) Analysis

The flavonoid content (FC) was assessed using an aluminum chloride-based colorimetric method, following the procedure described by Agbo et al. [31]. The absorbance was measured at 510 nm with a V-650 UV/Vis spectrophotometer**.** The FC was quantified as milligrams of quercetin equivalents (mg QE) per gram of OPD dry weight (g DW), based on a standard calibration curve prepared with quercetin.

#### 2.6.3. Total Betalain Analysis

The total content of betalains, including betaxanthins and betacyanins, was measured using a spectrophotometer (V-650, Jasco Inc., Easton, MD, USA). Absorbance was recorded at 480 nm for betaxanthins and 530 nm for betacyanins, following the method of Prakash and Manikandan [32]. Total betaxanthin content was expressed as milligram equivalents of indicaxanthin (mg IE) per gram of dry weight (g DW) of OPD, while total betacyanin content was expressed as milligram equivalents of betanin (mg BE) per gram of dry weight (g DW) of OPD, and results were computed by Equation (2):

BC(mg/L) = (A∙d_f_∙MW)/(ε∙L) (2)

where BC refers to either betacyanin or betaxanthin concentration, A is the absorbance measured (480 nm for betaxanthins, or 535 nm for betacyanins), MW represents the molecular weight (308,000 mg/mol for indicaxanthin or 550,000 mg/mol for betanin), ε denotes the molar extinction coefficient (60,000 L/mol·cm for betanin or 48,000 L/mol·cm for betaxanthin in water), d_f_ is the dilution factor, and L is the optical path length of the cuvette (1 cm).

#### 2.6.4. Determination of Antioxidant Capacity

The reducing antioxidant power (FRAP) assay

The ferric reducing antioxidant power (FRAP) assay of extracts was performed according to the method described by Benzie and Strain [33] with some modifications. The absorbance of the reacting mixture was measured at 593 nm with a V-650 UV/Vis spectrophotometer, and an ascorbic acid calibration curve was used. The FRAP values were expressed as mg of ascorbic acid equivalents (mg AAE) per gram of dry weight (g DW) of OPD.

The radical scavenging (DPPH) assay

The DPPH (1,1-diphenyl-2-picryl-hydrazil) radical scavenging activity of the extracts was evaluated as described by Fazio et al. [34], with some modifications. The scavenging activity was measured at 517 nm with a V-650 UV/Vis spectrophotometer. DPPH radical scavenging was represented as a percent inhibition (% inhibition) for the absorbance of the extract (Abs_sample_) with regard to the initial absorbance of DPPH (Abs_control_).

#### 2.6.5. Determination of the Anti-Inflammatory Activity

The anti-inflammatory activity of the OPD extracts was performed by hyaluronidase inhibition assay according to Gómez-Maqueo et al. [35]. The absorbance of the samples and blanks was measured with a spectrophotometer (SmartSpect Plus, BIOTable RAD, Hercules, CA, USA) at 400 nm. Results were expressed as the percentage of hyaluronidase inhibition (%).

#### 2.6.6. HPLC-DAD-ESI-Qtof Analysis of Bioactive Compounds

The individual betalains and phenolic compounds in the OPD extracts obtained from conventional SLE (control) and PEF pre-treated samples were analyzed by the method reported by Gómez-López et al. [12].

Briefly, OPD extracts were filtered with a 0.45 μm nylon filter (E0032, Análisis Vínicos, Tomelloso, Spain) and directly analyzed by HPLC-DAD and HPLC-DAD-ESI-Qtof. Concisely, a 1200 Series Agilent HPLC System (Agilent Technologies, Santa Clara, CA, USA) with a C18 reverse column (Zorbax SB-C18, 250 mm × 4.6 mm i.d., S-5 μm; Agilent, Barcelona, Spain) at 25 °C was employed. Phases A and B were carried out in a gradient over a period of 70 min using ultrapure water and formic acid (1% *v*/*v*) for phase A and methanol (99.8% LC-MS) and formic acid (1% *v*/*v*) for phase B. The injection volume was 20 µL, and the flow rate was 0.8 mL/min. The UV-visible photodiode array detector was set at 4 wavelengths: 280 nm for phenolic acids, 370 nm for flavonoids, 480 nm for betaxhantin, and 535 nm for betacyanins, simultaneously monitoring different chemical compound families. To confirm the chemical composition of each bioactive compound, HPLC-DAD-MS/Qtof and HPLC-DAD-ESI/MS were also conducted as reported by Gómez-López et al. [12].

### 2.7. Statistical Analysis

All the experiments, as well as the chemical and biological analysis, were performed at least in triplicate (n = 3). All data were expressed as mean ± standard deviation (SD), and SPSS Statistics Software 26.0 (IBM Corp., Armonk, NY, USA) was used to evaluate the data with one-way analysis of variance (ANOVA) followed by a post hoc Tukey’s test to determine the significant differences between the independent variables (*p* ≤ 0.05). The software Design Expert version 13 (Minneapolis, MN, USA) was used to determine the experimental design (FC-CCD), to analyze the data, and to define the optimal processing conditions that maximize both Zp and the extraction yields of the compounds of interest. The Pearson correlation coefficient (r) was calculated to determine the strength of the correlation between the response variables.

## 3. Results and Discussion

### 3.1. Physicochemical Characteristics of Opuntia Stricta var. Dillenii Whole Fruit (OPD)

All physicochemical characteristics of OPD whole fruit are shown in Table S1 (Supplementary Materials). This wild prickly pear variety is smaller than the cultivated ones, such as *Opuntia ficus-indica*, with a weight of around 54.33 ± 6.50 g. Another characteristic of these fruits is their low pH (3.82 ± 0.05) and titratable acidity of 1.46 ± 0.01 g citric acid/100 g fresh weight. The soluble solids (Brix°) of the whole fruit puree were 12.32 ± 0.60. Recent studies reported that OPD has lower soluble solids (Brix°) and pH and higher titratable acidity than other *Opuntia* spp. fruits, such as *Opuntia ficus-indica* fruits [1,12,14].

OPD whole fruit puree is characterized by its intense purple color (Figure 1). The color of the whole fruit puree was analyzed using CIELAB colorimeter parameters: 16.98 ± 1.27 L*, 15.96 ± 2.21 a*, and 3.09 ± 0.81 b* (Table S1, Supplementary Materials). The OPD fruit is a rich source of bioactive compounds, specifically betalains (betacyanins), which contribute to its purple color.

Optimization of SLE and PEF-assisted SLE was carried out using fresh whole fruit puree to promote optimal conductivity, ensure uniform treatment, minimize electrode fouling, and preserve sample integrity. Bioactive compound contents are reported based on the dry weight of the whole fruit to allow comparison with other previously assayed extraction processes. Data conversion was performed by calculating the water content of the puree (81.69%) from the difference between fresh and dry weight after freeze-drying of samples.

### 3.2. Optimization of Solid–Liquid Extraction (SLE) of Bioactive Compounds from OPD Whole Fruit Puree

#### 3.2.1. Bioactive Compounds and Biological Activities of SLE Extracts

**Figure 2:**
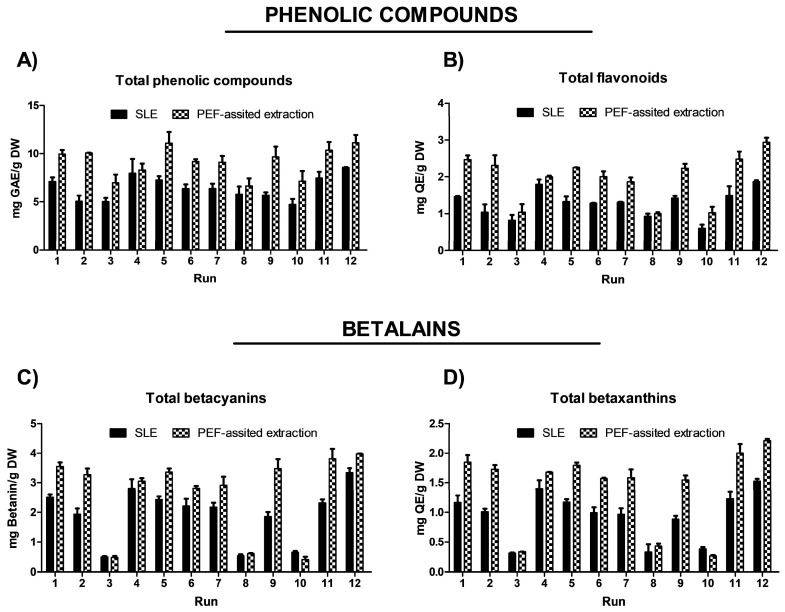
Experimental results of (**A**) TPC (mg GAE/g DW), (**B**) flavonoid content (mg QE/g DW), (**C**) total betacyanins (mg betanin/g DW), and (**D**) total betaxanthins (mg GAE/g DW) obtained from conventional SLE (Control) and PEF-assisted SLE process (E = 5 kV/cm; WT = 10.5 kJ/kg) from OPD whole fruit puree.

**Figure 3:**
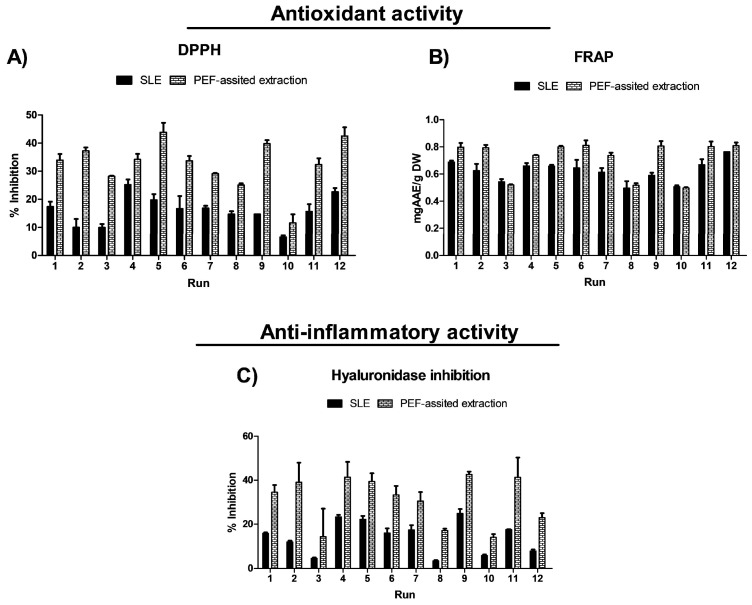
Experimental results of the biological activities (antioxidant (**A**,**B**) and anti-inflammatory (**C**)) of conventional SLE (control) and PEF-assisted (E = 5 kV/cm; WT = 10.5 kJ/kg) SLE from OPD whole fruit puree.

The impact of the three process variables (extraction time, temperature, and EtOH content in water) on the extraction efficiency of total phenolic compounds (TPC), total flavonoids (FC), and total betalain compounds (betacyanins and betaxanthins), as well as on the antioxidant and anti-inflammatory activities, is shown in Figure 2 and Figure 3 and data reported in Table S2 (Supplementary Materials).

In the obtained extracts, TPC ranged from 4.72 to 8.56 mg GAE/g DW. The highest TPC content was found when OPD puree was extracted with water as a solvent or with a water–ethanol mix (50%, *v*/*v*); meanwhile, the lowest amount was achieved with pure solvent (100% EtOH (*v*/*v*)) (runs 3, 8, and 10) (Figure 2A and Table S2, Supplementary Materials). These results suggested that pure organic solvent (ethanol) affects the extraction of phenolic compounds. More specifically, water and ethanol mixtures could enhance swelling of the fruit material, which facilitated mass transfer and improved extraction yields [36]. This effect aligns with some previous studies about the extraction of phenols and betalains from OPD using green technologies, such as ultrasound and pressurized liquid-assisted extraction [17]. OPD whole fruits are also rich in flavonoids. The SLE process results show that the total flavonoid content (FC) in the obtained extracts followed the same behavior observed for TPC (Figure 2B). The OPD extract obtained using water as the extraction solvent at 50 °C for 300 min (run 12) achieved 1.87 ± 0.04 mg QE/g DW, which was the highest content of total flavonoids (Table S2, Supplementary Materials).

The red to purple color of OPD fruits mainly derives from betacyanins, a class of pigments responsible for red-violet coloration with an absorption value of 535 nm [37]. However, some betacyanins, such as neobetanin, have a yellow to orange color and whose maximum absorption has a value of 467 nm, close to the betaxanthin (yellow color dye compounds) with an absorption value of 480 nm. Thus, in this study, the total betalain content was assessed by measuring total betacyanins at 535 nm and total betaxanthins at 480 nm. The content of total betalains in the extracts obtained by SLE processes from OPD ranged from 3.35 to 0.50 mg betanin/g DW and 1.53 to 0.31 mg indicaxanthin/g DW. Since betalains are water-soluble compounds, extractions using pure ethanol (runs 3, 8, and 10) yielded the lowest quantities (Figure 2C,D). Betalains are also thermolabile compounds, with their stability significantly declining between 50 and 80 °C. The more effective SLE extraction conditions involve the use of water as a solvent, a temperature of 50 °C, and an extraction time of 300 min (run 12), obtaining 3.35 ± 0.15 mg betanin/g DW and 1.53 ± 0.04 mg indicaxanthin/g DW (Table S2, Supplementary Materials).

The extraction process, temperature, time, and solvent significantly affect the extraction yield of bioactive compounds (Figure 2) as well as the antioxidant and anti-inflammatory activities of the obtained extracts (Figure 3). Antioxidant activity was measured using the ferric reducing antioxidant power (FRAP) assay and the radical scavenging assay (DPPH). The results are reported in Table S2 (Supplementary Materials) and illustrated in Figure 3. Extracts obtained from the SLE process in run 9 (50 °C, 300 min, and 50% ethanol in water (*v*/*v*)) show the highest antioxidant activity, 0.59 ± 0.02 mg AAE/g DW (FRAP) and 39.82 ± 1.22% reaction inhibition (DPPH) (Table S2, Supplementary Materials). Regarding the anti-inflammatory activity of the OPD extracts, the pattern was similar to that of antioxidant activity. Extracts from run 9 show the highest anti-inflammatory activity, with 24.90 ± 1.98% hyaluronidase inhibition.

#### 3.2.2. Model Fitting and RSM Analysis for the SLE Process

The data obtained from the FC-CCD design of the SLE extraction process, reported in Table 2, for all response variables were fitted to a second-order polynomial equation (Equation (2) in the Materials and Methods section). This second-order model was appropriate to define the influence of the independent variables [temperature (°C), time (min), and ethanol (EtOH) content in water (%, *v*/*v*)] on the target responses, including bioactive compound content and biological activities of the obtained extracts. The regression coefficients’ values, their significance, corresponding *p*-values, and the determination coefficients (R^2^) for each variable are reported in Table S3 (Supplementary Materials). The ANOVA results show determination coefficient (R^2^) values ranging from 0.991 to 1.000, indicating a robust correlation between observed and predicted data. Additionally, the analysis of variance confirmed the significance of the chosen model (*p* ≤ 0.05) for all responses, further supporting its predictive effectiveness (Table S3, Supplementary Materials).

**Figure 4:**
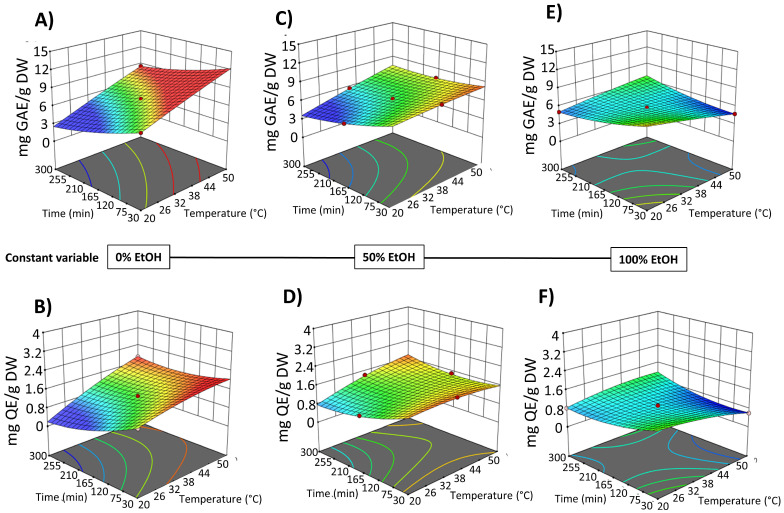
Response surfaces for TPC content (mg GAE/g DW) (**A**,**C**,**E**) and FC content (mg QE/g DW) (**B**,**D**,**F**) of the extracts obtained by SLE from OPD fruit, as a function of extraction time (min) and temperature (°C), with the ethanol in water (%, *v*/*v*) set at 0% (**A**,**B**), 50% (**C**,**D**), and 100% (**E**,**F**).

**Figure 5:**
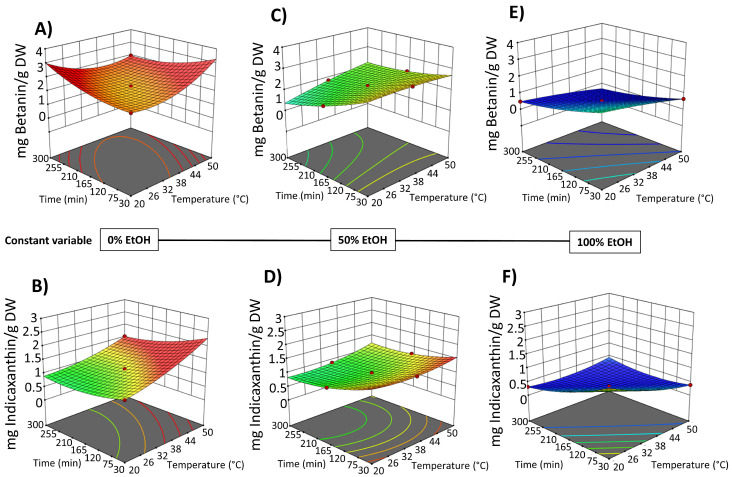
Response surfaces for total betacyanin content (mg betanin/g DW) (**A**,**C**,**E**) and total betaxanthin content (mg indicaxanthin/g DW) (**B**,**D**,**F**) of the extracts obtained by SLE from OPD fruit, as a function of extraction time (min) and temperature (°C), with the ethanol in water (%, *v*/*v*) set at 0% (**A**,**B**), 50% (**C**,**D**), and 100% (**E**,**F**).

The results indicated that in the SLE process from OPD whole fruit puree, the factor temperature did not exhibit a significant linear effect on most of the response variables, with a significant effect only on the extractability of TPC. However, EtOH content in water (as extraction solvent) and extraction time show a statistically significant effect on the extraction of bioactive compounds (Table S3, Supplementary Materials). Three-dimensional response surface graphs were employed to illustrate the effect of process variables on the solid–liquid extraction (SLE) of bioactive compounds from OPD whole fruit puree (Figure 4 and Figure 5). RSM was utilized to analyze the relationships between the extraction temperature (20–50 °C), extraction time (30–300 min), and EtOH content in water (0–100%, *v*/*v*). Figure 4 illustrates the relationship between the variables in the TPC and FC of the obtained extracts, while Figure 5 depicts the relationship between the variables and betalain (betacyanin and betaxanthin) content. These graphs revealed a linear negative trend with ethanol content in the extraction solvent for all bioactive compound extraction, indicating that the maximum response value decreases with increasing ethanol content across all responses, as Carpentieri et al. [30] reported in the TPC extraction from grape pomace. The amount of water increases the polarity of the extraction solvent, which accelerates the solvation of the bioactive compounds. Water also promotes the swelling of the matrix, facilitating the penetration of the solvent into the cell structure [38]. The observed negative linear trend can be explained by considering the polarity and solubility behavior of betalains and phenolic compounds from OPD, which are highly polar in nature. As the ethanol content increases, the overall polarity of the solvent system decreases, reducing the solubility and extraction efficiency of these hydrophilic compounds.

SLE process time also shows a negative significant effect on the bioactive compound content in the obtained extracts (Figure 4 and Figure 5). Extraction time is an essential variable in the extraction processes because it affects the efficiency of the recovery yield by increasing the contact between the solvent and the solid matrix. Short extraction times may result in partial extraction. However, if the extraction period is too long, the degradation of bioactive compounds may occur. Likewise, Prakash Maran and Manikandan [32], who investigated the effect of the extraction time (20–120 min) on the recovery yield of valuable intracellular compounds from *Opuntia ficus-indica*, demonstrated that the betaxanthin content initially increased with increasing the extraction time, which is probably due to the fact that the solvent can enter into the cells while more pigments can permeate into the solvent under the higher extraction time. Nevertheless, an extended extraction duration led to diminished phenolic content, as well as antioxidant activity and anti-inflammatory properties of the extracts. This decline is likely attributed to potential oxidation reactions or degradation phenomena, which may intensify at elevated extraction time [30]. The biological activity responses (antioxidant and anti-inflammatory activity) of the extracts obtained by SLE were also fitted to the second-order model (Table S3, Supplementary Materials) and show the same 3D trend as the bioactive compound content data (Figure S1, Supplementary Materials).

#### 3.2.3. Optimal Conditions for the SLE Process

Second-order polynomial models obtained in this study were utilized for each response in order to obtain specified optimum conditions. The Derringer’s desirability function method was used to optimize the process variables. This method converts each response into a desirability scale ranging from 0 to 1, with 0 indicating completely undesirable and 1 indicating fully desired responses [32]. In this case, the individual desirability was determined based on maximizing the extraction of bioactive compounds. The optimum parameter combination for green SLE was 45 °C, 8% EtOH content in water (*v*/*v*), and 128 min of extraction time. Under these conditions, the obtained extract shows a TPC of 8.67 ± 0.76 mg GAE/g DW, a flavonoid content of 1.55 ± 0.14 mg QE/g DW, a betacyanin content of 3.26 ± 0.36 mg betanin/g DW, and a betaxanthin content of 1.41 ± 0.27 mg indicaxanthin/g DW. Aligning with these optimum variables, Prakash Maran and Manikandan [32] concluded that the optimal conditions for solid–liquid extraction (SLE) of *Opuntia ficus-indica* pulp included an extraction temperature of 40 °C, a time of 115 min, and water as a solvent.

To characterize the individual betalain and phenolic compound content in the obtained extract by the optimal SLE conditions (45 °C, 8% EtOH content in water (*v*/*v*), and 128 min), an HPLC analysis was conducted.

#### 3.2.4. HPLC-DAD-ESI-Qtof Analysis of Bioactives in the SLE Extract from OPD Whole Fruit Puree

The resulting chromatogram profile and the content (mg/g dry weight (DW)) of the individual most abundant betalains and phenolic compounds are shown in Table 3 and Figure S2 (Supplementary Materials), respectively. The complete description of UV-vis and mass spectroscopy characteristics of all individual betalains and phenolic compounds found in OPD extracts was previously reported by Gómez López et al. [12]. The green extract obtained by the SLE process maintained the same betalain and phenolic compound profile as reported in the aforementioned study about the characterization of *Opuntia stricta* var. *dillenii* fruits and by-products.

As previously stated, OPD are an important source of betalains, mostly betacyanins (purple-colored dye compounds). Results reveal that optimized SLE from OPD whole fruit puree produced green extracts rich in betanin (peak 2), with 2.13 ± 0.49 mg/g DW. Additionally, it is a rich source of isobetanin (peak 3), 5″-O-E-sinapoyl-2′-apyosil-phyllocactin (peak 6), and neobetanin (peak 7), with a sum of all of them at 5.78 ± 0.36 mg/g DW (Table 3 and Figure S2, Supplementary Materials). While betanin is the most commonly found and studied betacyanin [39], other variations within the betacyanin class, such as isobetanin, phyllocactin, and neobetanin, also contribute to the overall pigmentation of OPD, as demonstrated in recent studies [12,40].

In addition to the betalains, the profile of phenolic compounds identified in OPD whole fruit extracts at wavelengths of 280 nm for phenolic acids and 370 nm for flavonoids is also shown in Figure S2 (Supplementary Materials). Among the phenolic acids, prickly pear fruits are rich in piscidic acid (peak 1), and it is accurate to say that this component is exclusive to certain plants such as *Opuntia* spp. [41]. In the optimal SLE process, an amount of 0.72 ± 0.31 mg/g DW was obtained (Table 3 and Figure S2, Supplementary Materials). Concerning flavonoids, the most predominant one was isorhamnetin-glucosyl-rhamnosyl-pentoside (IG2) at 0.11 ± 0.02 mg/g DW (peak 9).

To analyze if PEF pre-treatment enhanced the extractability of the bioactive compounds obtained using only the SLE process, a PEF-assisted SLE process was studied, carrying the optimization of the combined process in the same way as the previous data, with the optimization of the PEF pre-treatment the first step.

### 3.3. Optimization of the PEF-Assisted SLE Process of Bioactives from OPD

#### 3.3.1. Optimization of the PEF Pre-Treatment: Cell Membrane Disintegration Degree

The effect of the PEF pre-treatment on the degree of cell membrane disintegration (Zp) of OPD whole fruit puree was determined through complex electrical impedance measurements of untreated OPD puree and PEF-treated samples. This information was utilized to assess the Zp, which has been extensively proven to be a dependable marker of the extent of cell membrane permeabilization caused by PEF pre-treatment in various foods and agrifood by-products [29].

The constructed experimental design (FC-CCD) reported in Table 1 shows the effect of the independent variables, such as electric field strength (E, kV/cm) and energy inputs (Wt, kJ/kg), on the Zp of PEF-treated OPD tissue. The results demonstrated that an increase in the electric field strength and energy inputs led to a corresponding increase in Zp values. These findings agree with previous studies investigating the application of the PEF treatment on plant tissues such as microalgae, potatoes, tomato peels, and grape pomace [30,42]. Moreover, in the range of the investigated PEF treatment conditions, the effect of field strength appeared slightly more pronounced at fixed energy input than that of the energy input. The effect of the energy input, instead, appeared less significant when increasing the electric field strength applied from 3 kV/cm to 5 kV/cm. The highest Zp value of 0.57 ± 0.05 was observed at the highest PEF operating conditions (5 kV/cm and 20 kJ/kg) investigated.

The Zp values obtained in the current work are consistent with those reported by Surano et al. [24] for *Opuntia ficus-indica* fruits, which reached average Zp values of about 0.62 when applying PEF processing conditions of 1.05–1.4 kV/cm and 9.84 ± 2.08–12.59 ± 3.17 kJ/kg. The slightly lower values of Zp for OPD whole fruit puree can be likely attributed to the higher content of total fiber and fat (9.49 ± 1.51% and 0.71 ± 0.19%, respectively) compared to those found in *Opuntia ficus-indica* (5.37 ± 0.87% and 0.50 ± 0.13%, respectively), which can make the OPD whole fruit puree a more resistant sample to electro-permeabilization [2].

#### 3.3.2. Model Fitting and RSM Analysis for PEF Pre-Treatment

A quadratic polynomial equation (Equation (1), see Materials and Methods section) was chosen to model the data acquired from the experimental design (FC-CCD). The coefficients’ values and significance from the estimated polynomial model are detailed in Table S4 (Supplementary Materials). Findings revealed that the linear terms of both variables (electric field strength and energy input) significantly influenced (*p* ≤ 0.05) the permeability level of the plant tissue, while the interaction between individual variables was not significant (*p* > 0.05). Notably, the electric field strength emerged as the primary factor affecting the response variable, demonstrating a significant quadratic effect on Zp in contrast to the non-significant (*p* > 0.05) quadratic term for the energy input. The *p*-value of the model is less than 0.01, indicating its significance and corroborating that the independent variables exerted a substantial impact on the responses. Additionally, the model demonstrated a high level of adequacy with an R^2^ value of 0.9678, highlighting strong concordance between the experimental data and the predicted values.

**Figure 6:**
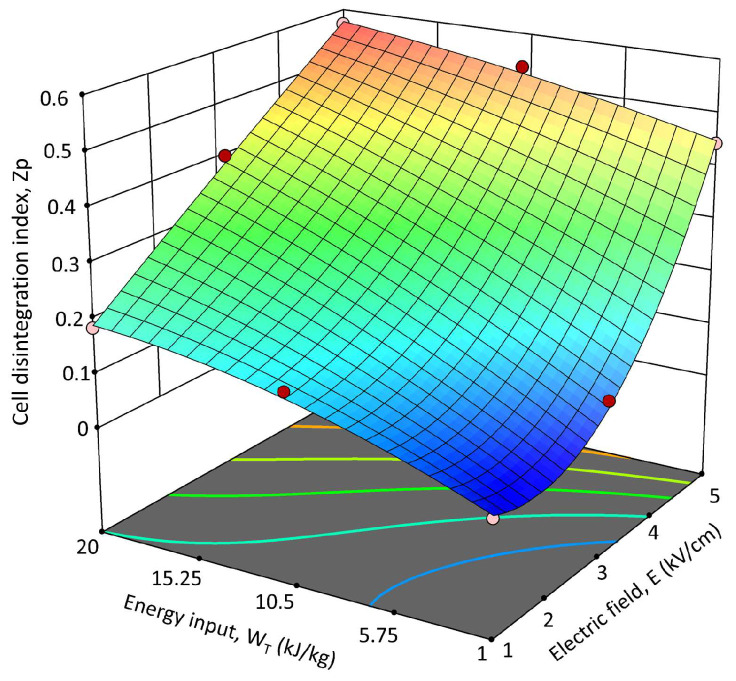
Response surface for the cell permeabilization index (Zp) of PEF-treated OPD whole fruit puree as a function of the electric field strength (E, kV/cm) and the energy input (WT, kJ/kg).

Figure 6 illustrates the 3D response surface graph that shows the interactions between field strength and energy input and how they affect the Zp of PEF-treated OPD whole fruit puree. The Zp shows a stronger dependency on the electric field strength than the one detected for the energy input, as previously discussed and better highlighted by the response surface graph (Figure 6). Other authors, who investigated the recovery of bioactive compounds such as phenolics or betalains from plant tissues (white grape pomace and prickly pear pulp) by applying PEF treatment, observed a similar behavior. According to the coefficients and significance associated with each factor of the model, the graph distinctly illustrates that Zp exhibited nearly linear growth with increasing energy input, while the field strength predominantly influenced the observed response in a quadratic way.

The optimal PEF pre-treatment conditions were determined based on the minimum electric field strength (Eopt, kV/cm) and energy input (WT-opt, kJ/kg) that resulted in the highest degree of Zp. The Zp value represents a significant index to select the optimal PEF pre-treatment conditions because electroporation was shown to be responsible for intensifying the release of bioactive compounds such as betalains and phenolic compounds in different plant materials, including prickly pears [27,42,43,44]. According to the obtained data, the maximum Zp value (0.571 ± 0.054) was achieved for 5 kV/cm and 20 kJ/kg. Nonetheless, there are no significant (*p* ≤ 0.05) differences between the Zp obtained when applying 5 kV/cm—10.5 kJ/kg and 5 kV/cm—20 kJ/kg (Table 1). Therefore, considering the lower WT, the optimal PEF pre-treatment conditions to be applied for maximizing the disintegration of OPD whole fruit puree were set at 5 kV/cm and 10.5 kJ/kg. These optimal parameters were employed to explore the impact of PEF pre-treatment application on the extractability of bioactive compounds from OPD whole fruit puree.

#### 3.3.3. Optimization of PEF-Assisted SLE of Bioactive Compounds from OPD

Bioactive compounds and biological activities of PEF-assisted SLE extracts

After the application of the PEF pre-treatment process at optimal conditions (electric field at 5 kV/cm and energy input at 10 kJ/kg) to the OPD whole fruit puree, the PEF-treated samples were submitted to the SLE extraction process. In the same way as the study conducted to optimize the SLE process (Section 3.2.) the impact of the three SLE process variables (extraction time, temperature, and EtOH content in water) on the extraction efficiency of total phenols (TPC), flavonoids (FC), and total betalain compounds, as well as on the antioxidant and anti-inflammatory activities of the obtained extracts, was investigated using FC-CCD design under response surface methodology. The experimental results are shown in Figure 2 for bioactive compound content and in Figure 3 for biological activities of the obtained extracts. The data are available in Table S5 (Supplementary Materials). The TPC ranged from 6.64 to 11.12 mg GAE/g DW for extracts obtained by the combined PEF-assisted SLE process, respectively (Table S5, Supplementary Materials). Extracts obtained by the combined PEF-assisted SLE process show an average of 1.4 times higher TPC yield compared to the extracts obtained from the SLE process without any pre-treatment (Figure 2). This increase in the extraction yield of total phenols could be attributed to the cell permeabilization induced by the electric field, which facilitates the release of phenolic compounds from intracellular to extracellular media [24]. Similar results were previously observed by other researchers, who found that higher electric fields increased the extraction of bioactive compounds, such as phenols and anthocyanins, from cell vacuoles [16,42,45,46]. As it was reported for SLE in the PEF-assisted SLE process, the highest TPC was found when OPD whole fruit puree was extracted with water as a solvent; meanwhile, the lowest amount was achieved with pure solvent (100% EtOH (*v*/*v*)), suggesting also that pure organic solvent negatively affects the extraction of phenolic compounds. The total flavonoids present in the extracts obtained by the PEF-assisted SLE process show similar behavior to the total phenolic compound content data. Extracts obtained using water as the extraction solvent at 50 °C for 300 min show the highest content of total flavonoids, with significant differences (*p* ≤ 0.05) between untreated and PEF pre-treated samples. The PEF-assisted SLE achieved 2.94 ± 0.13 mg QE/g DW (Table S5, Supplementary Materials). Therefore, an increment in total content of flavonoids (FC) of about 57% was observed in the extract of the PEF pre-treated sample combined with the SLE process in these conditions (Figure 2).

In the case of betalains, increasing the extraction temperature (up to 50 °C to prevent any degradation) and extending the extraction time using water as the solvent in the SLE extraction of pre-treated OPD puree with PEF had a significant positive effect on the extraction of betacyanins and betaxanthins, as clearly depicted in Table S5 (Supplementary Materials). For instance, Run 12, which involved OPD pre-treatment followed by SLE with water as the solvent for 300 min at 50 °C, yielded the highest amount of betalains, specifically a total content of betacyanins (3.97 ± 0.02 mg/g DW) and betaxanthin (2.21 ± 0.03 mg/g DW) (Figure 2 and Table S5, Supplementary Materials).

Regarding the antioxidant activities in general, PEF-assisted SLE enhances the antioxidant activity at least 2-fold higher than that of the extracts obtained by the conventional SLE (Figure 3). Moreover, in the present study, the bioactive compounds extracted show a positive Pearson correlation coefficient with the antioxidant activity determined by FRAP in the range of 0.96–0.98 for total betacyanins, 0.95–0.96 for total betaxhanthins, 0.84–0.91 for TPC, and 0.87–0.92 for FC. This data suggests that betalains and phenolic compounds mainly contribute to the overall antioxidant activity of the OPD extracts. On the contrary, the DPPH assay did not find improvement in antioxidant capacity in PEF pre-treated samples. Moreover, when the phytochemical content of the OPD extracts was correlated with the antioxidant activity determined by DPPH, a negative correlation was observed that ranged from 0.467 to 0.732 for TPC, FC, betacyanins, and betaxhantin responses, respectively. Antioxidant activity is a multifaceted property influenced by various factors beyond the presence of individual phytochemicals. While phytochemicals like phenolic acids, flavonoids, and betalains are known for their antioxidant properties, their effectiveness depends on their interaction with other compounds present in the sample. Other constituents in the samples or their interactions might offset the antioxidant potential, resulting in a negative correlation in the DPPH assay. The obtained data is consistent with the findings reported by Gómez-Maqueo et al. [35] in their study on the anti-inflammatory and antioxidant activities of prickly pear.

Regarding the anti-inflammatory activity of the OPD extracts obtained by PEF-assisted SLE, they exhibited twice the anti-inflammatory activity compared to the extracts obtained from the conventional SLE process (Figure 3). The conventional SLE process under different conditions produced extracts with 4.75 to 23.31% of hyaluronidase inhibition (Table S2), and the PEF-assisted SLE process produced OPD extracts with 14.02 to 42.66% of hyaluronidase inhibition (Table S5 in the Supplementary Materials). The data predicted that the use of less EtOH content in the solvent (water) (%, *v*/*v*) produced extracts with more anti-inflammatory activity. These results agreed with those obtained in the OPD extracts from ultrasound-assisted extraction (UAE) and pressurized-liquid extraction (PLE) using low EtOH content in water (%, *v*/*v*) [17]. This phenomenon can be attributed to the higher extraction of betalains at lower concentrations of ethanol, which correlates with the anti-inflammatory activity associated with these compounds [35]. In the present study, total betacyanin and total betaxanthin content were also strongly positively correlated with anti-inflammatory activity (r = 0.73–0.80).

In general, PEF pre-treatment can be considered a promising non-thermal technology for innovative extraction processes to obtain green extracts rich in betalains and phenolic compounds from OPD fruits, allowing for improved high-added-value compound yields with higher antioxidant and anti-inflammatory activities compared to the conventional SLE process.

Model fitting and RSM analysis of PEF-assisted SLE

The data obtained from the FC-CCD design of the PEF-assisted SLE process for all the response variables were fitted to a second-order polynomial equation (Equation (2) in the Materials and Methods section). This second-order model was appropriate to define the influence of the independent variables [temperature (°C), time (min), and ethanol (EtOH) content in water (%, *v*/*v*)] on the target responses, including bioactive compound content and biological activities of the obtained extracts. The regression coefficients’ values, their significance, corresponding *p*-values, and the determination coefficients (R^2^) for each variable are reported in Table S6 (Supplementary Materials).

The results show that, for OPD extracts obtained from the PEF-assisted SLE process, the temperature and time factors show a non-statistically significant linear effect in the SLE process applied after the PEF pre-treatment (Table S6 at Supplementary Materials). In this case as well, the EtOH content in water as a solvent (%, *v*/*v*) had a significant impact (*p* ≤ 0.05) on the extraction of bioactive compounds (Table S6, Supplementary Materials). Furthermore, the analysis of variance confirmed the significance of the chosen model (*p* ≤ 0.05) for all responses, providing further evidence of its predictive effectiveness. Three-dimensional response surface graphs were utilized to represent the effect of process variables on the extraction of bioactive compounds from OPD whole fruit puree.

**Figure 7:**
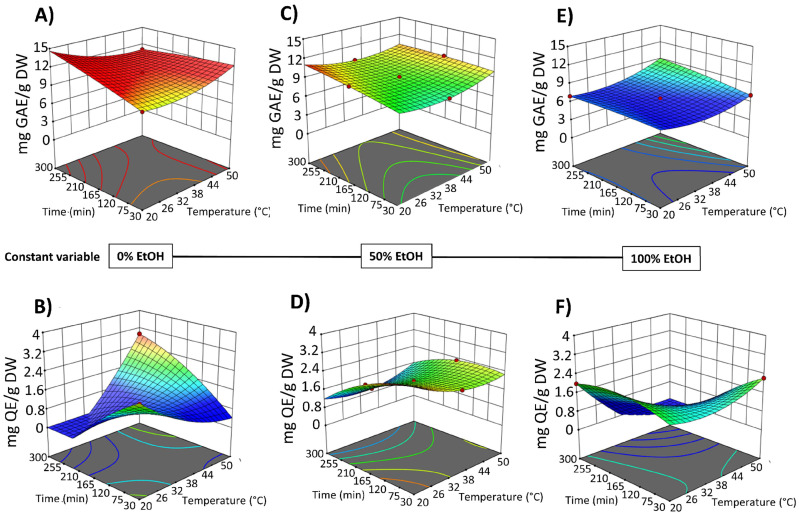
Response surfaces for TPC content (mg GAE/g DW) (**A**,**C**,**E**) and FC content (mg QE/g DW) (**B**,**D**,**F**) of the extracts obtained by PEF-assisted (E = 5 kV/cm; WT = 10.5 kJ/kg) SLE from OPD fruit, as a function of extraction time (min) and temperature (°C), with the ethanol in water (%, *v*/*v*) set at 0% (**A**,**B**), 50% (**C**,**D**), and 100% (**E**,**F**).

**Figure 8:**
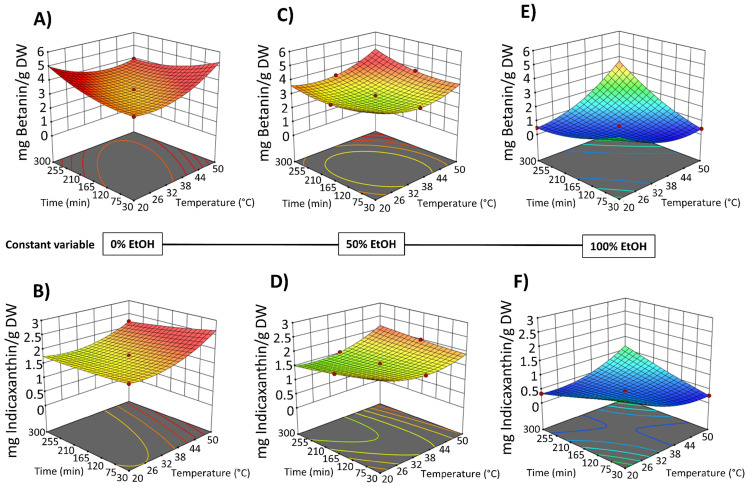
Response surfaces for total betacyanin content (mg betanin/g DW) (**A**,**C**,**E**) and total betaxanthin content (mg indicaxanthin/g DW) (**B**,**D**,**F**) of the extracts obtained by PEF-assisted (E = 5 kV/cm; WT = 10.5 kJ/kg) SLE from OPD fruit, as a function of extraction time (min) and temperature (°C), with the ethanol in water (%, *v*/*v*) set at 0% (**A**,**B**), 50% (**C**,**D**), and 100% (**E**,**F**).

Figure 7 shows the 3D graphs illustrating the effect of the variables on TPC and FC extraction, while Figure 8 shows the effect on betacyanins and betalains. It is evident that the extraction yield decreases as the concentration of EtOH in water increases, replicating the behavior observed in conventional SLE (with samples not pre-treated with PEF), as shown in Figure 2 and Figure 3. The negative linear and quadratic coefficients of the EtOH content in water (Table S2, Supplementary Materials) contributed to this observed trend. Similar trends were also observed by other researchers investigating ultrasound-assisted green extraction of betalains and phenolic compounds from OPD, red beet, and *Opuntia* spp. fruit peels [17,20,47].

Figure S3 (Supplementary Materials) shows the interaction between the examined independent SLE factors (temperature (°C), time (min), and EtOH content in water (%, *v*/*v*)) on the antioxidant (DPPH and FRAP) and anti-inflammatory (hyaluronidase inhibition) activity responses. The antioxidant activity of the extracts exhibited the same behavior as the TPC, FC, total betacyanins, and total betaxanthins. EtOH content in water (%, *v*/*v*) was the most impactful factor, and large concentrations of it significantly decreased the antioxidant activity (DPPH and FRAP) and anti-inflammatory activity of the extracts from PEF-assisted and (Figure S3, Supplementary Materials). For these responses, it was observed that the model coefficient values for EtOH content in water (%, *v*/*v*) were lower for PEF-assisted SLE samples (−9.39 and −0.1414) than for the conventional SLE process (−2.53 and −0.0807) (Tables S3 and S6 in the Supplementary Materials). This indicates that EtOH content in water (%, *v*/*v*) has a more negative effect on obtaining extracts with higher antioxidant activity in the PEF-assisted SLE process than in the conventional SLE process.

Optimal conditions for PEF-assisted SLE

An optimum condition for the PEF-assisted SLE process from OPD whole fruit puree was selected also using Derringer’s desirability function method, based on reported data. The individual desirability is determined based on maximizing the extraction of bioactive compounds. The optimum parameter combination was 35 °C, 0% ethanol in water (*v*/*v*), and 165 min of extraction for the PEF-assisted SLE process. With these conditions, the obtained extract shows a TPC of 12.35 ± 0.26 mg GAE/g DW, a flavonoid content of 1.97 ± 0.02 mg QE/g DW, a betacyanin content of 4.91 ± 0.41 mg betanin/g DW, and a betaxanthin content of 2.28 ± 0.20 mg indicaxanthin/g DW.

In the SLE extraction process, the optimal conditions were 45 °C, 8% EtOH content in water (*v*/*v*), and 128 min extraction time. Therefore, the application of PEF prior to SLE can successfully increase the extraction yields (ranging up by 27 to 68%) of bioactive compounds from OPD, enhance the antioxidant and anti-inflammatory properties of the extracts, and reduce the temperature of the extraction process. Continuously, experiments were conducted using the optimal PEF pre-treatment operating conditions (Eopt = 5 kV/cm; WT, opt = 10.5 kJ/kg) at optimal SLE (35 °C, 0% EtOH content in water (*v*/*v*), and 165 min) to compare with the optimal conditions for conventional SLE (45 °C, 8% EtOH content in water (*v*/*v*), and 128 min) in order to examine the impact of PEF pre-treatment on the individual phenolic and betalain extraction by HPLC-DAD following the methodologies reported in a recent publication [12].

#### 3.3.4. HPLC-DAD-ESI-Qtof Analysis of Bioactive Compounds of OPD Extracts

Table 4 shows the individual bioactive compound content (mg/g DW) and extraction yield (%) analyzed by HPLC and biological activities (antioxidant and anti-inflammatory) of the OPD whole fruit puree extracts obtained through PEF-assisted SLE under optimum conditions (35 °C, 165 min, and water as a solvent). The PEF-SLE process maintained the same betalain and phenolic compound profile reported by Gómez-López et al. [12] of *Opuntia stricta* var. *dillenii* fruits and by-products. Figure S2 (Supplementary Materials) shows the HPLC-DAD chromatograms of major betalains and phenolic compounds in OPD fruit extracts. PEF pre-treatment enhanced the extractability of the bioactive compounds compared to the optimized SLE process (without any pre-treatment). The extraction yield was calculated by comparing the bioactive compounds extracted by SLE (control) (Table 3) with those extracted by PEF-assisted SLE (Table 4).

The application of PEF pre-treatment caused a significant (*p* > 0.05) increment in betanin (by 41%), isobetanin (by 51%), 2′-O-apiosyl-4-O-phyllocactin (by 96%), 5″-O-E-sinapoyl-2′-apyosil-phyllocactin (by 99%), and neobetanin (by 54%) extraction contents (Table 4). Kouba et al. [27] and López et al. [45] reported that PEF pre-treated beetroot produced extracts with an increased extraction yield of betalains. Overall, after PEF pre-treatment, there was no degradation of betalains; in contrast, the yield increased for all betalain compounds. The sum of the identified betalains increased from 5.78 ± 0.36 mg/g DW (control) to 9.32 ± 0.63 mg/g DW, with a 61% increment (Table 4). In accordance with these findings, the ultrasound-assisted “green” extraction (UAE) of betalains and phenolic compounds from OPD whole fruit demonstrated that at the optimum UAE conditions (at 15% ethanol in solvent, 50% amplitude, and 20 °C), 10.06 mg of total major betalains/g DW were obtained [17]. It is remarkable that PEF-assisted SLE from OPD produced quite similar outcomes without requiring the use of ethanol in the solvent mixture.

Regarding the phenolic compounds, PEF pre-treatment results in a 135% increase in piscidic extraction, obtaining values of 1.70 ± 0.01 mg/g DW. These results are comparable to those obtained [17] in the study of the application of pressurized green liquid extraction to obtain green extracts from OPD whole fruit (lyophilized material), which obtained 2.08 mg/g DW [17]. Concerning flavonoids, PEF pre-treatment enhanced the extractability of isorhamnetin glucox-yl-rhamnosyl-pentoside (IG2) more than twice (by 117%). Summarizing, the total phenolic compound extraction (the sum of the piscidic acid and flavonoids) was enhanced (by 138%) using the PEF pre-treated SLE in comparison to the conventional process (SLE), which uses ethanol (8%) as an extraction solvent (Table 4).

## 4. Conclusions

The proposed model significantly predicted the optimum extraction conditions for both conventional solid–liquid extraction (SLE) and pulsed electric field-assisted SLE (PEF-assisted SLE) processes from *Opuntia stricta* var. *dilenii* (OPD) whole fruit puree. For PEF-assisted SLE, the optimal parameters include a PEF pre-treatment with an energy input (WT) of 10.5 Kj/kg and an electric field strength (E) of 5 kV/cm, followed by SLE extraction at 35 °C for 165 min with 0% ethanol in water (*v*/*v*). Conversely, for SLE without PEF, the optimal conditions involve extraction at 45 °C with 8% ethanol content in water (*v*/*v*) for 128 min. HPLC analysis confirmed that PEF pre-treatment of OPD whole fruit fresh puree increased the subsequent SLE yield of the sum of identified betalains by up to 61% and the extraction yield of the sum of identified phenolic compounds (phenolic acids and flavonoids) by up to 135%. The optimal PEF-assisted SLE produced extract with an identified total betalain content of 9.32 ± 0.63 mg/g dry weight, a piscidic acid content of 1.70 ± 0.11 mg/g dry weight, and a major total flavonoid content of 0.32 ± 0.03 mg/g dry weight. No evidence of degradation of individual compounds was reported after PEF pre-treatment. Additionally, PEF pre-treatment enhanced the antioxidant activity (by 145% for DPPH and by 28% for FRAP) and anti-inflammatory activity (by 19% hyaluronidase inhibition) compared with conventional SLE without pre-treatment. Overall, the use of PEF pre-treatment before SLE leads to obtaining extracts richer in betalains and phenolic compounds with higher biological activities, along with the use of lower temperatures and ethanol in the solvent (%, *v*/*v*), reducing costs and energy consumption.

## Figures and Tables

**Table 1:** FC-CCD design of the two independent variables (E and WT) and the response of the dependent variable (Zp) of the PEF-treated OPD whole fruit puree.

Run	Variables		Response
	E (kV/cm)	W_T_ (kJ/kg)	Zp
1	1	1	0.013 ± 0.001 ^a^
2	1	10.5	0.147 ± 0.007 ^bc^
3	1	20	0.185 ± 0.009 ^bc^
4	3	1	0.080 ± 0.005 ^ab^
5	3	10.5	0.238 ± 0.012 ^c^
6	3	20	0.399 ± 0.021 ^de^
7	5	1	0.444 ± 0.058 ^ef^
8	5	10.5	0.535 ± 0.061 ^fg^
9	5	20	0.571 ± 0.054 ^g^

Superscript letters indicate statistically significant differences (*p* ≤ 0.05).

**Table 2:** FC-CCD design of the three independent variables: temperature (°C), time (min), and ethanol in water (%, *v*/*v*) used for the SLE step in the PEF-assisted or non-assisted extraction process.

Run Number	Factor X1: Temperature (°C)	Factor X2: Time (min)	Factor X3: Ethanol in Water (%)
1	20	30	0
2	20	165	50
3	20	300	100
4	35	30	50
5	35	165	0
6	35	165	50
7	35	165	50
8	35	165	100
9	35	300	50
10	50	30	100
11	50	165	50
12	50	300	0

**Table 3:** Bioactive compound content (mg/g DW) of the major betalains and phenolic compounds analyzed by HPLC and biological activities (antioxidant and anti-inflammatory) of the OPD extracts obtained through SLE under optimum conditions (45 °C, 128 min, and 8% ethanol in water).

Peak *	Compound	Family	mg/g DW
1	Piscidic acid	Phenolic acid	0.72 ± 0.31 ^A^
2	Betanin	Betalain	2.13 ± 0.49 ^A^
3	Isobetanin	Betalain	0.84 ± 0.14 ^A^
4	2′-O-apiosyl-4-O-phyllocactin	Betalain	0.52 ± 0.04 ^A^
5	5″-O-*E*-sinapoyl-2′-apyosil-phyllocactin	Betalain	1.11 ± 0.06 ^A^
6	Neobetanin	Betalain	1.18 ± 0.18 ^A^
7	Quercetin-3-O-rhamnosyl-rutinoside (QG3)	Flavonoid	n.d.
8	Quercetin glycoside(QG2)—Quercetin hexose pentoside	Flavonoid	0.01 ± 0.00 ^A^
9	Isorhamnetin glucoxyl-rhamnosyl-pentoside (IG2)	Flavonoid	0.11 ± 0.02 ^A^
	Sum of major betalains		5.78 ± 0.36 ^A^
	Sum of major flavonoids		0.12 ± 0.03 ^A^
	Biological activities		
	Antioxidant activity		
	FRAP	mg AAE/g DW	0.72 ± 0.03 ^A^
	DPPH	% of inhibition	15.31 ± 0.77 ^A^
	Anti-inflammatory activity		
	Hyaluronidase inhibition	% of inhibition	31.76 ± 4.40 ^A^

Results were expressed as mean ± standard deviation (n = 5). This came from obtaining at least two independent extracts (n = 2) and performing HPLC determinations of each time (n = 3). Superscript capital letters indicate statistically significant differences (*p* ≤ 0.05) between obtained extracts assisted by PEF-assisted SLE (Table 4). n.d. No detected; * peak according to Figure S2.

**Table 4:** Bioactive compound content (mg/g DW) and extraction yield (%) of the major betalains and phenolic compounds analyzed by HPLC and biological activities (antioxidant and anti-inflammatory) of the OPD extracts obtained through PEF-assisted SLE under optimum conditions (35 °C, 165 min, and water as a solvent).

Peak *	Compound	Family	PEF-Assisted SLE	Increment Yield
			(mg/g DW)	(%)
1	Piscidic acid	Phenolic acid	1.70 ± 0.11 ^B^	+136%
2	Betanin	Betalain	3.00 ± 0.01 ^B^	+41%
3	Isobetanin	Betalain	1.26 ± 0.03 ^B^	+50%
4	2′-O-apiosyl-4-O-phyllocactin	Betalain	1.02 ± 0.03 ^B^	+96%
5	5″-O-*E*-sinapoyl-2′-apyosil-phyllocactin	Betalain	2.21 ± 0.64 ^B^	+99%
6	Neobetanin	Betalain	1.82 ± 0.01 ^B^	+54%
7	Quercetin-3-O-rhamnosyl-rutinoside (QG3)	Flavonoid	0.02 ± 0.00 ^B^	+100%
8	Quercetin glycoside(QG2)—Quercetin hexose pentoside	Flavonoid	0.06 ± 0.01 ^B^	+150%
9	Isorhamnetin glucoxyl-rhamnosyl-pentoside(IG2)	Flavonoid	0.24 ± 0.02 ^B^	+118%
	Sum of major betalains		9.32 ± 0.63 ^B^	+61%
	Sum of major flavonoids		0.32 ± 0.03 ^B^	+166%
	Biological activities			
	Antioxidant activity			
	FRAP	mg AAE/g DW	0.92 ± 0.01 ^B^	+28%
	DPPH	% of inhibition	37.61 ± 0.01 ^B^	+146%
	Anti-inflammatory activity			
	Hyaluronidase inhibition	% of inhibition	45.18 ± 1.12 ^B^	+118%

Results were expressed as mean ± standard deviation (n = 5). This came from obtaining at least two independent extracts (n = 2) and performing HPLC determinations of each time (n = 3). Superscript capital letters indicate statistically significant differences (*p* ≤ 0.05) between obtained extracts by SLE (Table 3). n.d. No detected; * peak according to Figure S2. Yield was calculated comparing conventional SLE with PEF-assisted SLE.

## Data Availability

Data are contained within the article and Supplementary Materials. Further inquiries can be directed to the corresponding authors.

## Supplementary Materials

The following supporting information can be downloaded at: https://www.mdpi.com/article/10.3390/foods14172934/s1, Table S1. Physicochemical characteristics of OPD from the Canary Islands (Spain); Table S2. Bioactive compound content and biological activities for the extract obtained at different SLE variable combinations (FC-CCD); Table S3. Analysis of variance (ANOVA) of the second polynomial models for responses in OPD extracts from conventional SLE; Table S4. Analysis of variance (ANOVA) of the second polynomial model describing the influence of PEF processing parameters (E and WT) on the cells disintegration index (Zp) of OPD whole fruit puree; Table S5. Bioactive compound content and biological activities for the extract obtained by PEF-assisted SLE (Eopt = 5 kV/cm; WT-opt = 10.5 kJ/kg) at different SLE variable combinations (FC-CCD); Table S6. Analysis of variance (ANOVA) of the second polynomial models for responses in OPD extracts from PEF-assisted SLE; Figure S1. Response surfaces for biological activity (antioxidant and anti-inflammatory) of the extracts obtained by SLE from OPD whole fruit puree; Figure S2. HPLC-DAD chromatogram of major betalains and phenolic compounds in OPD fruit extracts obtained from untreated (control) SLE at optimum conditions (at 45 °C, 128 min, and 8% ethanol in water) and PEF-treated SLE at optimum conditions (at 35 °C, 165 min, and water as a solvent) and extraction processes; Figure S3. Response surfaces for biological activity (antioxidant and anti-inflammatory) of the extracts obtained by PEF-assisted (E = 5 kV/cm; WT = 10.5 kJ/kg) SLE from OPD whole fruit puree.

## Abbreviations

The following abbreviations are used in this manuscript:

DW	Dry Weight
E	Electric Field
EtOH	Ethanol
FC	Total Flavonoid Content
FC-CCD	Face-Centered Central Composite Design
HPLC	High-Performance Liquid Chromatography
OPD	*Opuntia stricta* var. *dillenii*
PEF	Pulsed Electric Fields
QE	Quercetin Equivalent
RSM	Response Surface Methodology
SLE	Solid–Liquid green Extraction
TPC	Total Phenolic Compounds
WT	Energy Input
Zp	Cell Disintegration Index
