# Supplementary material for: Pulsed Electric Field-Assisted “Green” Extraction of Betalains and Phenolic Compounds from Opuntia stricta var. dillenii Prickly Pears: Process Optimization and Biological Activity of Green Extracts
Source: Foods. 2025 Aug 22;14(17):2934. doi: 10.3390/foods14172934 (PMC12427632; doi:10.3390/foods14172934)
Supplement: Supplementary file 1 [file foods-14-02934-s001.zip › foods-3786800-supplementary.pdf]

Pulsed Electric Fields assisted "green" extraction of betalains and phenolic compounds from *Opuntia stricta* var. *dillenii* prickly pears: Process optimization and biological activity of green extracts

Supplementary Material

Table S1. Physicochemical characteristics of OPD from Canary Island (Spain).

**Physicochemical characteristics**	***OPD* whole fruit**
Weight (g)	54.33 ± 6.50
Soluble solids (ºBrix)	12.32 ± 0.60
pH	3.42 ± 0.01
Tiritable acidity (g citric acid/100 g fresh weight)	1.46 ± 0.01
Moisture (%)	81.69 ± 0.56
Color (CIELab)	
*L (lighteness)**	16.98 ± 1.27
*a (greennes-redness)**	15.96 ± 2.21
*b (blueness-yellowness)**	3.09 ± 0.81

Table S2. Bioactive compounds content and biological activities for the extract obtained at different SLE variable combinations (FC-CCD).

RUN	Bioactive compounds extraction							Biological activities		
				TPC	FC	Total betacyanins	Total betaxanthins	Antioxidant activity		Anti-inflammatory
	Temperature °C	Time *min*	Ethanol in water *%, v/v*	*mg GAE/g DW*	*mg QE/g DW*	*mg BE/g DW*	*mg IE/g DW*	FRAP *mg AEE/g DW*	DPPH *% inactivation*	Hyaluronidase *% inhibition*
**1**	20	30	0	7.09 ± 0.44^{Acd}	1.46 ± 0.02^{Acd}	2.51 ± 0.10^{Abc}	1.17 ± 0.12^{Ab}	0.69 ± 0.01^{Ade}	33.92 ± 2.17^{Bbcd}	15.82 ± 0.47^{Abc}
**2**	20	165	50	5.06 ± 0.59^{Abc}	1.04 ± 0.21^{Abc}	1.94 ± 0.19^{Abc}	1.01 ± 0.05^{Aab}	0.62 ± 0.05^{Acd}	37.18 ± 1.18^{Bcd}	11.90 ± 0.59^{Abc}
**3**	20	300	100	5.02 ± 0.39^{Aab}	0.82 ± 0.15^{Aab}	0.50 ± 0.03^{Aa}	0.31 ± 0.01^{Aa}	0.54 ± 0.02^{Aabc}	28.24 ± 0.12^{Bbc}	4.57 ± 0.32^{Aa}
**4**	35	30	50	7.98 ± 1.46^{Ade}	1.80 ± 0.13^{Acd}	2.81 ± 0.31^{Abc}	1.40 ± 0.14^{Ab}	0.66 ± 0.02^{Acde}	34.21 ± 1.94^{Bcd}	23.31 ± 0.93^{Ac}
**5**	35	165	0	7.25 ± 0.39^{Acd}	1.33 ± 0.14^{Abcd}	2.43 ± 0.11^{Abc}	1.17 ± 0.05^{Ab}	0.66 ± 0.01^{Acde}	43.85 ± 3.38^{Be}	22.16 ± 1.60^{Acd}
**6**	35	165	50	6.37 ± 0.44^{Acd}	1.28 ± 0.01^{Abcd}	2.22 ± 0.24^{Abc}	1.00 ± 0.09^{Aab}	0.64 ± 0.06^{Acd}	33.67 ± 1.74^{Bcd}	15.91 ± 2.15^{Abc}
**7**	35	165	50	6.38 ± 0.50^{Acd}	1.31 ± 0.01^{Abcd}	2.17 ± 0.15^{Abc}	0.97 ± 0.10^{Aab}	0.61 ± 0.03^{Abcd}	29.09 ± 0.26^{Bbc}	15.43 ± 1.63^{Abc}
**8**	35	165	100	5.80 ± 0.80^{Abcd}	0.93 ± 0.07^{Aab}	0.54 ± 0.05^{Aa}	0.33 ± 0.13^{Aa}	0.50 ± 0.05^{Aa}	25.06 ± 0.56^{Bb}	3.38 ± 0.41^{Aa}
**9**	35	300	50	5.70 ± 0.29^{Abcd}	1.43 ± 0.06^{Acd}	1.86 ± 0.15^{Ab}	0.88 ± 0.06^{Aab}	0.59 ± 0.02^{Abcd}	39.82 ± 1.22^{Bde}	24.90 ± 1.98^{Ac}
**10**	50	30	100	4.72 ± 0.58^{Aa}	0.60 ± 0.10^{Aa}	0.66 ± 0.04^{Aa}	0.38 ± 0.03^{Aa}	0.51 ± 0.01^{Aab}	11.61 ± 3.03^{Aa}	5.80 ± 0.51^{Aab}
**11**	50	165	50	7.48 ± 0.64^{Ade}	1.50 ± 0.25^{Acd}	2.32 ± 0.12^{Abc}	1.23 ± 0.12^{Ab}	0.67 ± 0.04^{Ade}	32.34 ± 2.26^{Bbcd}	17.50 ± 0.17^{Abcd}
**12**	50	300	0	8.56 ± 0.04^{Ae}	1.87 ± 0.04^{Ad}	3.35 ± 0.15^{Ac}	1.53 ± 0.04^{Ab}	0.76 ± 0.00^{Ae}	42.50 ± 3.10^{Bde}	7.82 ± 0.68^{Aab}

Results were expressed as mean ± standard deviation (n = 4). This came from obtaining at least two independent extracts (n=2). Superscript capital letters indicate statistically significant differences ($p \leq 0.05$) between obtained extract assisted by PEF and only SLE (Table S5). Superscript lowercase letters indicate statistically significant differences ($p \leq 0.05$) between runs for the same extraction mode.

Table S3. Analysis of variance (ANOVA) of the second polynomial models for responses in OPD extracts from conventional SLE.

**Coefficients**	**Bioactive compounds extraction**						
	**SLE**						
	TPC	FC	Total betacyanins	Total betaxanthins	Antioxidant activity		Anti-inflammatory
	mg GAE/g DW	*mg QE/g DW*	*mg Betanin/g DW*	*mg Indicaxanthin/g DW*	*DPPH % inactivation*	*FRAP mg AEE/g DW*	*Hyaluronidase % inhibition*
β_0	6.52	1.36	2.14	1.04	35.66	0.7817	39.19
A-Temperature	1.21 *	0.2267 ns	0.1884 ns	0.1087 ns	-2.47 ns	0.0048 ns	1.12 *
B-Time	-1.14 *	-0.1867 *	-0.473 *	-0.2586 *	0.8089 *	0.0246 *	3.14 **
C-EtOH	-0.7267 *	-0.1991 *	-0.9453 **	-0.4201 *	-9.39 *	-0.1414 *	-8.17 **
AB	0.7528 *	0.2774 *	0.229 ns	0.0784 ns	-0.25 *	0.0058 ns	-0.88 *
AC	-1.59 **	-0.343 *	-0.6434 **	-0.3308 *	-5.49 *	0.0164 ns	5.95 **
BC	0.9180 *	0.1793 ns	-0.0621 ns	0.0008 ns	-0.46 ns	0.0075 ns	4.06 **
A^2	-0.3218 ns	-0.12 ns	0.0114 ns	0.0574 ns	-1.96 ns	0.0171 ns	-1.90 **
B^2	0.2519 ns	0.2233 *	0.2174 *	0.0789 ns	-1.76 ns	-0.0199 ns	2.43 **
C^2	-0.0623 ns	-0.2606 *	-0.632 **	-0.3106 *	-2.32 ns	-0.1229 *	-16.82 **
***p-value* of the model**	0.0198 *	0.0401 *	0.0052 **	0.0277 *	0.0302 *	0.0357 *	0.0043 **
R^2	0.9956	0.991	0.9988	0.9938	0.9998	0.9919	1.000

(*) Means that are significant for $p \le 0.05$, (**) significant for $p \le 0.01$ and (ns) no significant for $p > 0.05$

Table S4. Analysis of variance (ANOVA) of the second polynomial model describing the influence of PEF processing parameters (E and WT) on the cells disintegration index (Zp) of OPD whole fruit puree.

**Coefficients**	***Zp*** Cell disintegration index
β_0	0.02490
A-Energy field (E)	0.2008 ***
B-Energy input (W_T)	0.1030 **
AB	0.011 [ns]
A^2	0.0808 *
B^2	0.0208 [ns]
***p-value* of the model**	0.0044 **
**R^2**	0.9678

(*) Means that are significant for $p \leq 0.05$, (**) significant for $p \leq 0.01$, (***) significant for $p \leq 0.001$, and ([ns]) no significant for $p > 0.05$

Table S5. Bioactive compounds content and biological activities for the extract obtained by PEF-assisted (Eopt = 5 kV/cm; WT-opt = 10.5 kJ/kg) at different SLE variable combinations (FC-CCD).

**RUN**	Bioactive compounds extraction							Biological activities		
				TPC	FC	Total betacyanins	Total betaxanthins	Antioxidant activity		Anti-inflammatory
	Temperature °C	Time *min*	Ethanol in water *%, v/v*	*mg GAE/g DW*	*mg QE/g DW*	*mg BE/g DW*	*mg IE/g DW*	*FRAP mg AEE/g DW*	*DPPH % inactivation*	*Hyaluronidase % inhibition*
**1**	20	30	0	9.93 ± 0.45^{Bcd}	2.47 ± 0.11^{Bcd}	3.55 ± 0.14^{Bbc}	1.85 ± 0.12^{Bbc}	0.80 ± 0.03^{Bb}	17.43 ± 1.65^{Aabc}	34.35 ± 3.34^{Bb}
**2**	20	165	50	10.05 ± 0.05^{Bcd}	2.31 ± 0.27^{Bbc}	3.27 ± 0.21^{Bbc}	1.73 ± 0.07^{Bb}	0.79 ± 0.02^{Bb}	10.06 ± 2.92^{Aab}	39.07 ± 8.88^{Bbc}
**3**	20	300	100	6.97 ± 0.85^{Bab}	1.04 ± 0.22^{Aa}	0.48 ± 0.05^{Aa}	0.34 ± 0.00^{Ba}	0.52 ± 0.00^{Aa}	10.04 ± 1.13^{Aab}	14.27 ± 1.27^{Ba}
**4**	35	30	50	8.30 ± 0.66^{Abcd}	2.00 ± 0.03^{Abc}	3.05 ± 0.10^{Ab}	1.68 ± 0.01^{Bb}	0.74 ± 0.00^{Bb}	25.26 ± 1.76^{Ac}	41.38 ± 6.94^{Bc}
**5**	35	165	0	11.09 ± 1.14^{Bd}	2.25 ± 0.01^{Bbc}	3.36 ± 0.12^{Bbc}	1.79 ± 0.05^{Bbc}	0.80 ± 0.01^{Bb}	19.78 ± 2.08^{Aab}	39.44 ± 3.67^{Bbc}
**6**	35	165	50	9.18 ± 0.24^{Bbcd}	2.00 ± 0.15^{Bbc}	2.81 ± 0.08^{Ab}	1.57 ± 0.01^{Bb}	0.81 ± 0.04^{Bb}	16.69 ± 4.51^{Aab}	33.35 ± 4.08^{Bb}
**7**	35	165	50	9.10 ± 0.66^{Bbcd}	1.87 ± 0.11^{Bb}	2.91 ± 0.29^{Ab}	1.58 ± 0.14^{Bb}	0.74 ± 0.02^{Bb}	16.84 ± 0.84^{Aab}	32.39 ± 3.78^{Bb}
**8**	35	165	100	6.64 ± 0.79^{Aa}	1.00 ± 0.04^{Aa}	0.61 ± 0.02^{Aa}	0.43 ± 0.04^{Aa}	0.52 ± 0.02^{Aa}	14.71 ± 0.99^{Aab}	17.10 ± 0.87^{Bab}
**9**	35	300	50	9.66 ± 1.07^{Bbcd}	2.24 ± 0.12^{Bbc}	3.48 ± 0.32^{Bbc}	1.54 ± 0.08^{Bb}	0.81 ± 0.04^{Bb}	14.75 ± 0.07^{Aab}	42.66 ± 0.23^{Bc}
**10**	50	30	100	7.14 ± 1.06^{Bbc}	1.03 ± 0.16^{Ba}	0.41 ± 0.09^{Aa}	0.27 ± 0.01^{Ba}	0.50 ± 0.01^{Aa}	6.55 ± 0.63^{Aa}	14.02 ± 1.44^{Ba}
**11**	50	165	50	10.36 ± 0.84^{Bd}	2.48 ± 0.21^{Bcd}	3.81 ± 0.33^{Bc}	2.00 ± 0.15^{Bbc}	0.80 ± 0.04^{Bb}	15.63 ± 2.62^{Aabc}	41.31 ± 4.03^{Bbc}
**12**	50	300	0	11.12 ± 0.80^{Bd}	2.94 ± 0.13^{Bcd}	3.97 ± 0.02^{Bc}	2.21 ± 0.03^{Bc}	0.81 ± 0.02^{Bb}	22.67 ± 1.36^{Ab}	22.99 ± 2.01^{Bab}

Results were expressed as mean ± standard deviation (n = 4). This came from obtaining at least two independent extracts (n=2). Superscript capital letters indicate statistically significant differences ($p \leq 0.05$) between obtained extract assisted by PEF and only SLE (Table S2). Superscript lowercase letters indicate statistically significant differences ($p \leq 0.05$) between runs for the same extraction mode.

Table S6. Analysis of variance (ANOVA) of the second polynomial models for responses in OPD extracts from PEF-assisted SLE.

**Coefficients**	**Bioactive compounds extraction**						
	**PEF-SLE**						
	TPC	FC	Total betacyanins	Total betaxanthins	Antioxidant activity		Anti-inflammatory
	mg GAE/g DW	*mg QE/g DW*	*mg Betanin/g DW*	*mg Indicaxanthin/g DW*	*DPPH % inactivation*	*FRAP mg AEE/G DW*	*Hyaluronidase % inhibition*
β_0	9.42	1.63	3.13	1.65	17.41	0.6297	18.80
A-Temperature	0.155 ^{ns}	-0.0853 ^{ns}	0.2706 ^{ns}	0.137 ^{ns}	2.78 *	0.0221 ^{ns}	2.8 ^{ns}
B-Time	0.679 ^{ns}	-0.6271 ^{ns}	0.2149 ^{ns}	-0.0678 ^{ns}	-5.26 **	-0.0347 *	0.7924 ^{ns}
C-EtOH	-2.22 **	0.0076 *	-1.38 *	-0.6807 *	-2.53 *	-0.0807 *	-9.39 *
AB	-0.4868 ^{ns}	0.3015 *	0.2803 ^{ns}	0.1837 ^{ns}	3.34 *	0.0303 **	-6.07 *
AC	0.4246 *	-0.6856 *	0.092 ^{ns}	-0.1767 ^{ns}	-7.44 *	-0.0531 **	3.1 ^{ns}
BC	-0.1858 ^{ns}	-0.263 ^{ns}	0.1821 ^{ns}	0.0632 ^{ns}	2.35 ^{ns}	0.0027 ^{ns}	4.49 *
A^2	0.6688 *	0.9401 ^{ns}	0.2895 ^{ns}	0.1805 ^{ns}	-4.88 *	0.0244 ^{ns}	-5.67 ^{ns}
B^2	-0.5603 ^{ns}	-0.1716 *	0.0115 ^{ns}	-0.0738 ^{ns}	2.28 ^{ns}	-0.0025 *	3.74 *
C^2	-0.6762 *	-0.4201 *	-1.27 *	-0.5729 *	-0.48 ^{ns}	-0.0448 **	-7.79 ^{ns}
***p-value* of the model**	0.0114 *	0.0285 *	0.0499 *	0.0176 *	0.0221 *	0.0459 *	0.0341 *
**R^2**	0.9916	0.9999	0.9887	0.9961	0.995	0.9896	0.9988

(*) Means that are significant for $p \leq 0.05$, (**) significant for $p \leq 0.01$ and (^{ns}) no significant for $p > 0.05$

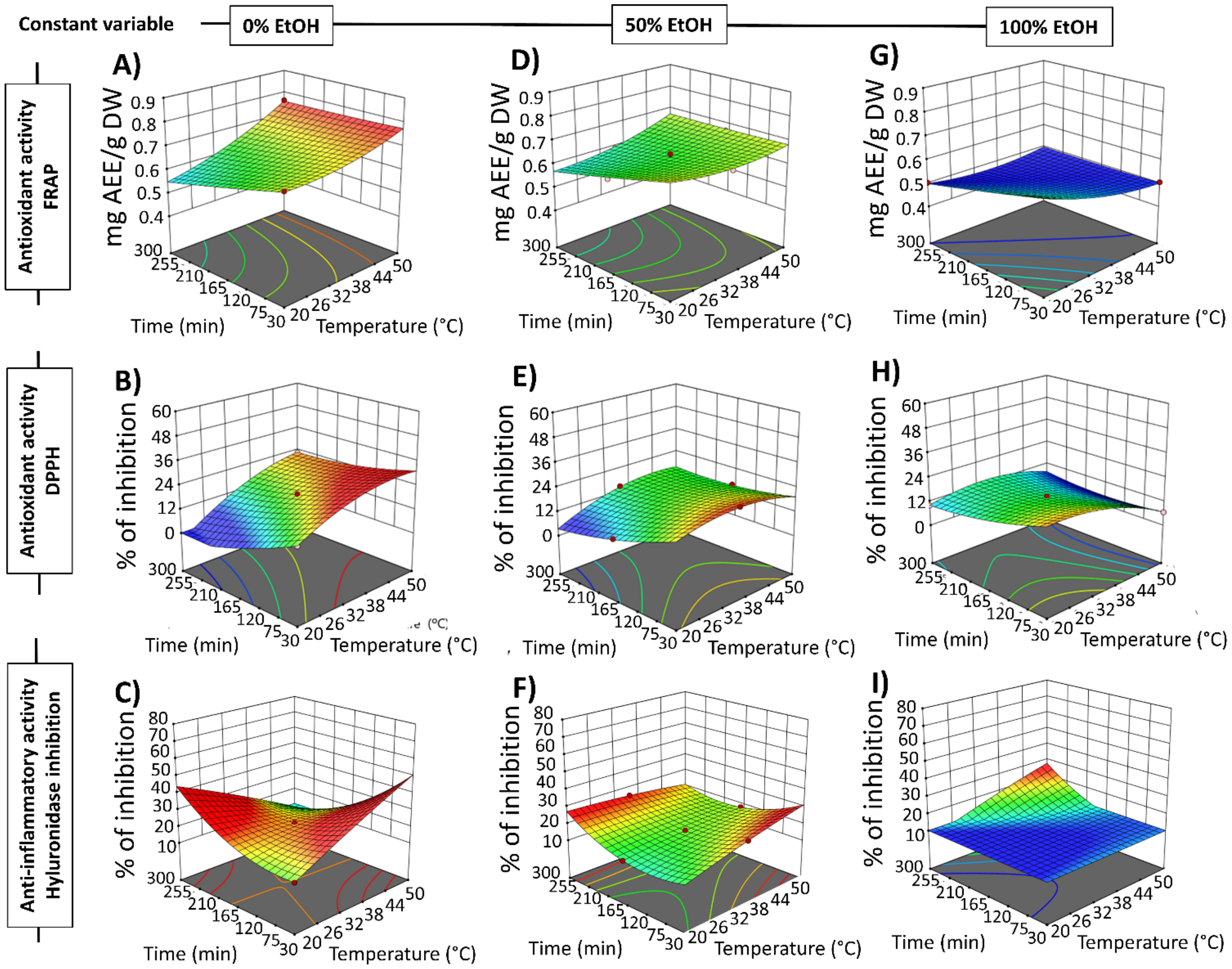

Figure S1. Response surfaces for biological activity (Antioxidant and anti-inflammatory) of the extracts obtained by SLE from OPD whole fruit puree.

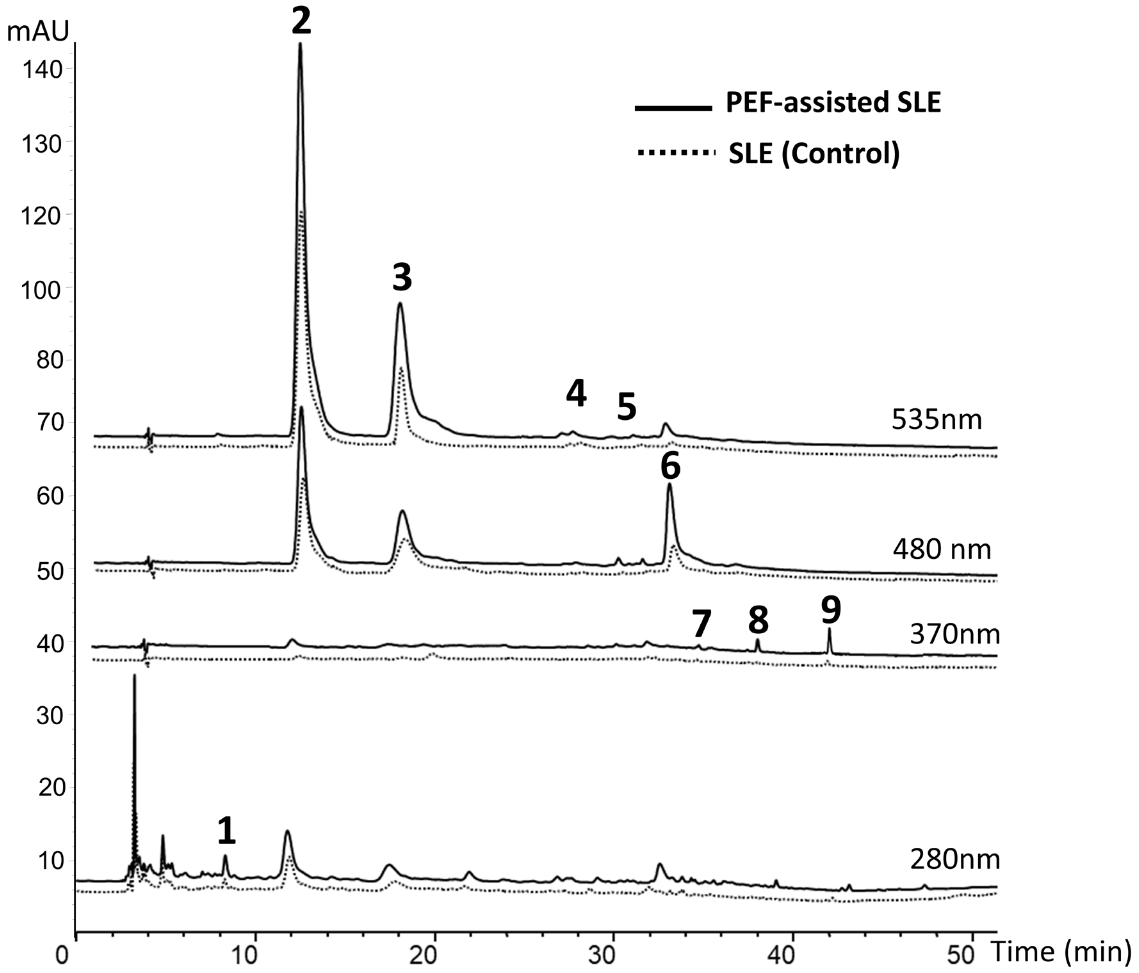

Figure S2 HPLC-DAD chromatogram of major betalains and phenolic compounds in OPD fruit extracts obtained from untreated (control) SLE optimum conditions (at 45°C, 128 minutes, and 8% ethanol in water) and PEF-treated SLE at optimum conditions (at 35°C, 165 minutes, and water as a solvent) extraction processes.

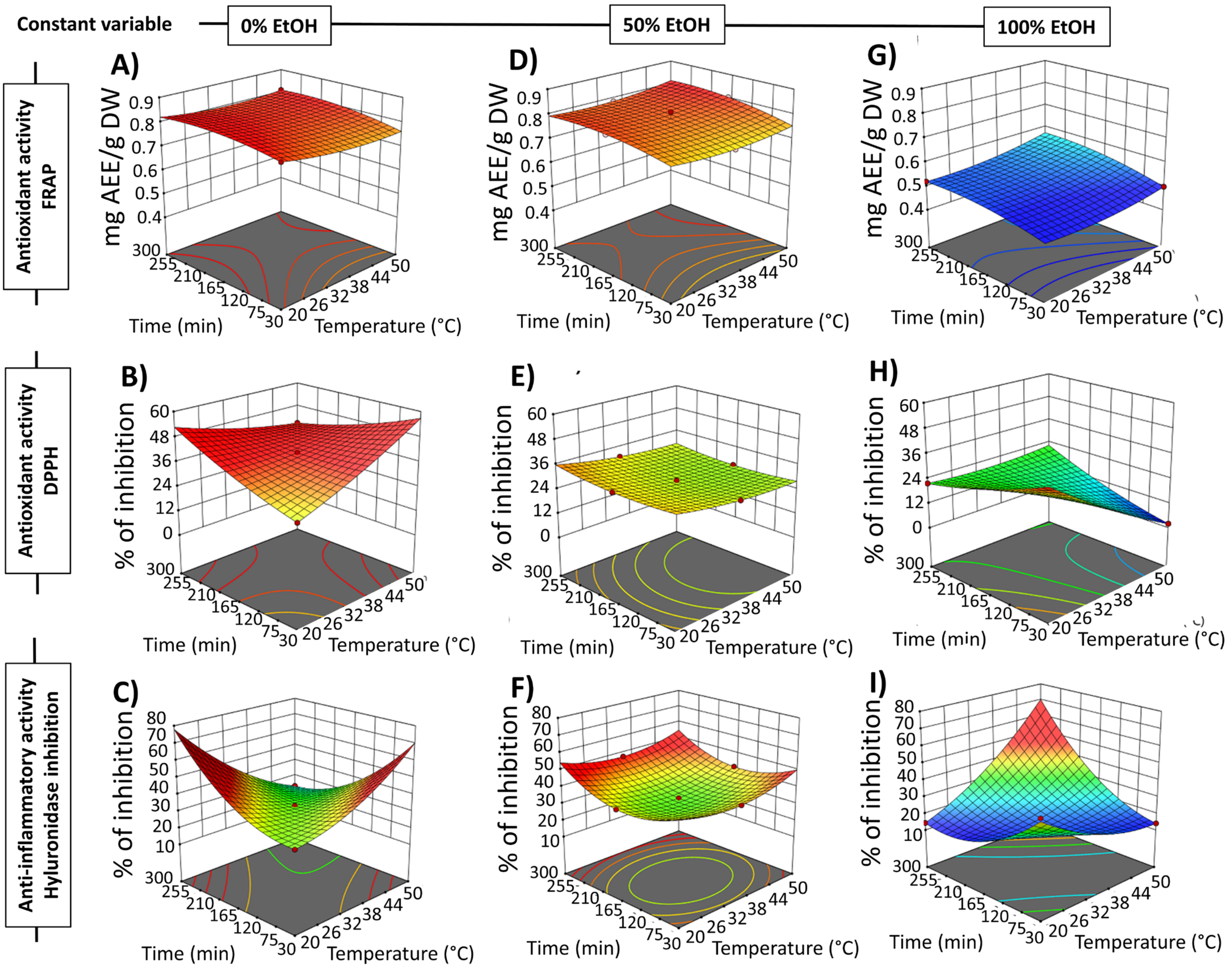

Figure S3. Response surfaces for biological activity (Antioxidant and anti-inflammatory) of the extracts obtained by PEF-assisted (E = 5 kV/cm; WT = 10.5 kJ/kg) SLE from OPD whole fruit puree.
